# Global Health Estimates: Stronger Collaboration Needed with Low- and Middle-Income Countries

**DOI:** 10.1371/journal.pmed.1001005

**Published:** 2010-11-30

**Authors:** Osman Sankoh

**Affiliations:** INDEPTH Network, Accra, Ghana

## Abstract

Osman Sankoh argues for much stronger collaboration between generators of global health estimates, and individuals and organizations working at the country level, as part of a *PLoS Medicine* cluster of articles on global health estimates.

**Summary Points**

- The United Nations and other international agencies should continue to generate global health indicators through their traditional sources of data.
- Stronger collaboration is needed, however, with southern-based organisations such as the INDEPTH Network in the generation of the estimates and determining how they should be published.
- In the absence of effective vital registration systems in many low- and middle-income countries, data generated by health and demographic surveillance systems could strengthen global estimates, especially in the area of directly measured cause of death statistics.

*This article is part of a cluster of five articles on global health estimates.*

Traditionally the United Nations (UN) and its specialised agencies (including the World Health Organization [WHO], the United Nations Children's Fund [UNICEF], and the United Nations Statistics Division), as well as the World Bank and the United States Center for Disease Control and Prevention, have generated estimates of health indicators. For many countries, especially low- and middle-income countries (LMICs) where civil registration systems are not effective for various reasons, these estimates have been the only credible outputs available to policy-makers and planners—yet significant questions remain as to the authenticity of their underlying data sources [1].

Recent controversies about whether UN agencies or academics should make such estimates, and how they should be published, are timely and important [2],[3]. Unfortunately, however, southern-based health organisations are conspicuous for their absence in these scenarios. Whoever compiles estimates, all institutions involved should strengthen collaboration with researchers, policy-makers, and organizations at the grass-roots level in LMICs.

One southern organisation trying to make a difference in this respect is the INDEPTH Network (http://www.indepth-network.org), an umbrella for currently more than 40 discrete population surveillance centres in LMICs in Africa, Asia, and Oceania. Such sources have the potential to complement and strengthen both the data from traditional providers to the UN system and the publicly available data that are used by others. Here, in addition to other data sources, the INDEPTH Network is explored as an example of how health and demographic surveillance system (HDSS) data could strengthen global estimates, especially in the area of directly measured cause of death statistics. HDSSs enumerate and longitudinally follow up populations in geographically well-defined areas to record demographic and health changes that occur over time. More specifically, they collect detailed information on fertility, mortality, and migrations within those populations.

## Ineffective Civil Registration Systems in Many LMICs

In the absence of complete vital registration in many LMICs, WHO/UN and other international agencies have depended on national censuses covering entire populations, usually conducted every ten years. Other sources have included demographic and health surveys and periodic national cross-sectional surveys such as the United Nations Children's Fund Multiple Indicator Cluster Surveys and the World Bank Living Standards Measurement Study surveys. While these surveys provide nationally representative indicators, their cross-sectional nature makes them incapable of following up individuals and households over a long period of time as HDSSs do. HDSS data have been used to produce life tables for LMICs based on empirical data [4], and INDEPTH Network studies have been an important source of data for estimating regional and global disease burdens and setting priorities [5]–[8]. In a recent article, Bangha et al. [9] described the potential role that can be played by the INDEPTH Network in monitoring the UN Millennium Development Goals [MDGs].

## Generating Cause of Death Statistics

Cause of death statistics are the most difficult to generate in most LMICs. In rural communities the great majority of deaths occur at home without an attending physician or even a recent consultation with a physician. The INDEPTH Network uses a standardised method of verbal autopsy to provide vital information on cause of death in these communities [10]. INDEPTH Network data contributed to WHO's “The Global Burden of Disease: 2004 Update” [11]. This is an important area where a stronger collaboration with the INDEPTH Network and other southern-based organisations that collect health and demographic data will lead to more credible data for global estimates.

## Conclusion

Despite the reservations about global health estimates [1], the UN and other international agencies should continue to generate these health indicators through their traditional sources of data—censuses, demographic and health surveys, Multiple Indicator Cluster Surveys, Living Standards Measurement Study surveys—or from data generated by health care institutions. They should, however, strengthen collaboration with other LMIC-based organisations. Hill et al. [12] consider all approaches used to provide necessary information about population health as “interim” so long as these are not part of fully effective civil registration systems. AbouZahr et al. [13] lament that “over the past 30 years, the global health and development community has failed to provide the needed technical and financial support to countries to develop civil registration systems….Each country faces a different set of challenges, and strategies must be tailored accordingly.” Given the dearth in resources in many LMICs, it seems appropriate that WHO/UN should continue generating estimates of health statistics in these countries if those statistics are to maintain the credibility they have had over the years irrespective of the apparent challenges about the sources of the information. Bangha et al. [9] state that “[w]hat is needed now is to harness the potential that INDEPTH HDSS centres offer, together with other national level data, to inform national programmes monitoring the achievement of MDGs.” I would add that WHO/UN should play a role in encouraging LMICs to establish HDSSs and, in sourcing funds for HDSSs, to generate more vital information.

## Abbreviations

HDSS: health and demographic surveillance system
LMIC: low- and middle-income country
MDG: Millennium Development Goal
WHO: World Health Organization
